# Why Would the Brain Need Dormant Neuronal Precursors?

**DOI:** 10.3389/fnins.2022.877167

**Published:** 2022-04-08

**Authors:** Bruno Benedetti, Sebastien Couillard-Despres

**Affiliations:** ^1^Institute of Experimental Neuroregeneration, Paracelsus Medical University, Salzburg, Austria; ^2^Spinal Cord Injury and Tissue Regeneration Center Salzburg, Paracelsus Medical University, Salzburg, Austria; ^3^Austrian Cluster for Tissue Regeneration, Vienna, Austria

**Keywords:** neuronal precursors, neuronal maturation, neurogenesis, cognition, neuronal plasticity

## Abstract

Dormant non-proliferative neuronal precursors (dormant precursors) are a unique type of undifferentiated neuron, found in the adult brain of several mammalian species, including humans. Dormant precursors are fundamentally different from canonical neurogenic-niche progenitors as they are generated exquisitely during the embryonic development and maintain a state of protracted postmitotic immaturity lasting up to several decades after birth. Thus, dormant precursors are not pluripotent progenitors, but to all effects extremely immature neurons. Recently, transgenic models allowed to reveal that with age virtually all dormant precursors progressively awaken, abandon the immature state, and become fully functional neurons. Despite the limited common awareness about these cells, the deep implications of recent discoveries will likely lead to revisit our understanding of the adult brain. Thus, it is timely to revisit and critically assess the essential evidences that help pondering on the possible role(s) of these cells in relation to cognition, aging, and pathology. By highlighting pivoting findings as well as controversies and open questions, we offer an exciting perspective over the field of research that studies these mysterious cells and suggest the next steps toward the answer of a crucial question: why does the brain need dormant neuronal precursors?

## Introduction

Dormant non-proliferative neuronal precursors (dormant precursors) are a particular type of undifferentiated immature neuron that resides in several cortical and subcortical areas of the adult mammalian brain, outside the canonical neurogenic niches (Bonfanti, 2013; König et al., 2016). These dormant precursors are characterized by the same markers of immaturity than precursors generated through adult neurogenesis, which has contributed to some confusion in the field. However, the origin and fate of these cells are fundamentally different (Feliciano et al., 2015).

## Do We Need Dormant Precursors?

Regrettably, for almost three decades, awareness about dormant precursors has been limited due the predominant focus placed on canonical adult neurogenesis (Bonfanti and Seki, 2021): on one side, the complex roles and relevance of canonical neurogenesis in the adult brain were intensively investigated and yet, are still under debate (Bonfanti and Amrein, 2018; Sorrells et al., 2021). On the other, the existence of dormant precursors have been proposed since the late nineties, but did not receive as much attention and became a matter of increasing interest only recently (Feliciano et al., 2015). A possible explanation for the lower awareness about dormant precursors is the observation of an age-associated decline in the number of these precursors, combined with the lack of reliable methods to trace their ultimate fate. Until not long ago, it was not possible to clarify whether dormant precursor progressively matured and became undistinguishable from other surrounding neurons, or whether they died as result of their lack of integration (Bonfanti and Seki, 2021). In other words, were dormant precursors relevant or were they vestigial?

Very recent breakthroughs demonstrated the progressive maturation and functional integration of dormant precursors as excitatory principal neurons in the murine olfactory cortex (Rotheneichner et al., 2018; Benedetti et al., 2019). These reports brought more attention on dormant precursors and shifted perspectives in regard to the adaptive and regenerative capacities of the adult brain (Feliciano et al., 2015; Palazzo et al., 2018; Parolisi et al., 2018; Seki, 2020; Sorrells et al., 2021). Therefore, we will first briefly summarize some crucial discoveries. After the first indication provided by a seminal work (Klempin et al., 2011), a definitive evidence of dormant precursors maturation and integration in the adult brain came thanks to the availability of novel transgenic mice in which the expression of fluorescent reporters can be permanently induced in dormant precursors (Zhang et al., 2010; Rotheneichner et al., 2018). This model based on the doublecortin (DCX) promoter allows to permanently label the immature neurons, such as the dormant precursors of the olfactory cortex, at specific ages and trace their fate in the brain thereafter, i.e., beyond the time at which the expression of immaturity markers has waned. By these means, the maturation of dormant precursors in the olfactory cortex was unequivocally documented.

In young adults, most immature dormant precursors are small, endowed with few short neurites. They express the immaturity markers DCX and polysialylated-neural cell adhesion molecule (PSA-NCAM), receive a very sparse synaptic input, and have neither axons nor the capacity of repetitive action potential firing. Such cells are strictly postmitotic in the adult brain and are generated by proliferative processes during embryonic development according to patterns of BrdU labeling (Gómez-Climent et al., 2008; Piumatti et al., 2018; Rotheneichner et al., 2018). Throughout adulthood, the expression of immaturity markers wanes and dormant precursors awaken slowly and in staggered fashion, in response to yet unknown stimuli. When the maturation which was stalled since embryonic age finally resumes, precursors develop larger soma, longer and more branched dendrites, spines and synapses, and *a bona fide* axon. Former dormant precursors become capable of repetitive action potential firing and receive increasing amount of synaptic input (Rotheneichner et al., 2018; Benedetti et al., 2019; Coviello et al., 2021). Finally, in the aged brain, former dormant precursors have become neurons that express markers, such as NeuN, and remain thereafter in the brain for the whole lifespan of an individual. Current research that addresses aspects of dormant precursor maturation in more brain areas, as well as aspects of connectivity including pre- and postsynaptic targets will soon provide more insight on these neurons.

**FIGURE 1:**
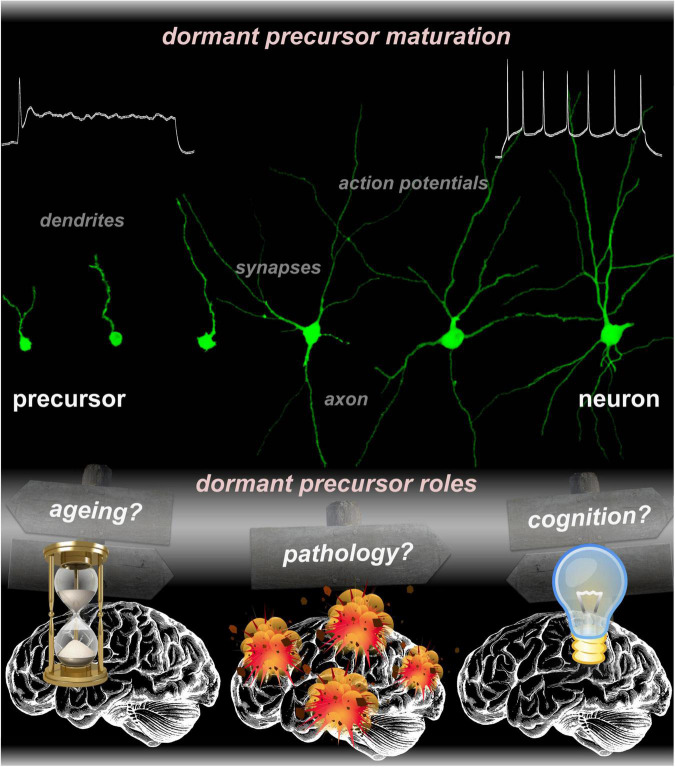
Micrographs collection representing various states of dormant precursors undergoing progressive maturation, from an inactive to a fully functional neuron. The function(s) of dormant precursors is still elusive. Current research across multiple species suggested potential roles in aging, pathology, and cognition.

While the long-standing dilemma over the capacity of the adult brain to integrate dormant precursors and make use of them has been irrefutably resolved, it is timely to revisit the potential function(s) of dormant precursors (Figure 1). Thus, it is pertinent to question the function of dormant precursors to help finding pivoting points for the research that will answer another crucial, yet unresolved matter: Why would the brain need dormant precursors?

## When, Where, and Which Dormant Precursors Do We Need?

Sub-populations of dormant precursors exhibit distinctive markers, suggesting that these cells constitute a heterogeneous pool and that may fulfill multiples roles. For example, dormant precursors of cortical and subcortical areas of several species mature into GABAergic interneurons (Dayer et al., 2005; Ponti et al., 2008; Cai et al., 2009; Rubio et al., 2016). At the same time, other dormant precursors mature into principal glutamatergic neurons that often, but not always, occupy the cortical layer II of several brain areas (Gómez-Climent et al., 2011; Varea et al., 2011; Rubio et al., 2016; Rotheneichner et al., 2018; Sorrells et al., 2019). Thus, even if dormant precursors are often grouped within a single class, they are pre-determined to generate excitatory or inhibitory neurons that most likely have completely different roles according to the species and the brain area in which they mature. Despite the apparent heterogeneity of dormant precursors, their abundance in the adult brain of longevous gyrencephalic species is highly suggestive for their relevance during adulthood (La Rosa et al., 2020).

It should be noted however, that the availability of dormant precursors over the whole lifespan, could result in a different function for these cells according to the age of their integration. Hence, on one hand, some dormant precursors will integrate into the juvenile brain, whereas, on the other, some dormant precursors will maintain immaturity for decades, as shown in a remarkable study on the human brain (Sorrells et al., 2019). Taken that dormant precursors integrate in the young, adult and perhaps even the aged brain, for what age would their integration be most relevant? To tackle this question, one could consider what evolutionary advantage may derive from being equipped with these cells during adulthood, as well as what are the possible reasons for the residual immaturity of some precursors in the aged brain.

## Why Do We Need Dormant Precursors?

### Cognition

A cross-species comparison of dormant precursor distribution in the adult mammalian brain showed the highest abundance in the cerebral cortex of gyrencephalic species (La Rosa et al., 2020). Additionally, it is worth noting that precursors spread across many cortical areas and appear to be particularly enriched in secondary processing layers. Therefore, dormant precursors may be evolutionary relevant for cognition and higher order processing (Palazzo et al., 2018; La Rosa et al., 2020; La Rosa and Bonfanti, 2021). Even in mice, a species where dormant precursors are less represented, these cells are most abundant in the olfactory cortex layer II, with increasing gradient from primary to secondary sensory areas (Gómez-Climent et al., 2008; Luzzati et al., 2009; Klempin et al., 2011; Rossi et al., 2014; König et al., 2016; Rubio et al., 2016; Rotheneichner et al., 2018; Benedetti et al., 2019).

In small-brained, lissencephalic species such as rodents, olfaction is a preponderant sensory input driving higher-order processing (Shepherd and Rowe, 2017). In mice, olfactory learning controls central aspects of adult behavior related to habitat exploration, avoidance of predators, nesting, and reproduction-related behavior (DeLong, 1967; Alyan and Jander, 1994; Alyan, 1996; Kemble and Bolwahnn, 1997; Nevison et al., 2000; Latham and Mason, 2004). For an adult mouse, critical olfactory-based choices are guided by specific cues that undergo constant changes (e.g., abundance of food, partners, or predators). Integration of dormant precursors may adapt and optimize processing of sensory inputs from salient individual experiences and situations at the level of circuit connectivity activated during adulthood. In this context, noradrenaline which controls different aspects of network plasticity involved in olfactory learning (Sullivan and Wilson, 1994; Rangel and Leon, 1995; Harley, 2004; Moreno et al., 2012) might be able to modulate the maturation of dormant precursors (Vadodaria et al., 2017). A role for additional monoamines in dormant precursor maturation has also been suggested (Coviello et al., 2020). Consequently, learning and adaptation in several adult brain regions devoid of neural stem cells might imply the introduction of new neurons arising through awakening of dormant precursors, possibly driven by the processing of salient experiences.

Notwithstanding caveats of translating inferences across species (Bonfanti and Amrein, 2018), one could hypothesize that integration of dormant precursors in different areas of the neocortex also accounts for improved multimodal sensory processing along adulthood in gyrencephalic mammals (Aboitiz and Montiel, 2015; La Rosa et al., 2020). Humans may even use dormant precursors to better manage their highly variable ecosystems, not only in terms of time and space, but also culture, language, and patterns of social interactions. However, the link between dormant precursors and behavior still remains very speculative as the only known attempt, to the best of our knowledge, to explore the direct relation between dormant precursors and behavior did not lead to conclusive evidence (Gómez-Climent et al., 2011). Thus, evidence over an association between altered precursor maturation and behavioral impairments remains so far indirect (Avino et al., 2018; Sorrells et al., 2019).

### Aging

Could the dormant precursors be necessary to assure a healthy brain aging? A critical detractor of this hypothesis is the evidence that few precursors retain immaturity until the brain is aged. On the contrary, most dormant precursors are maturing during adolescence and early adulthood (Rotheneichner et al., 2018; Benedetti et al., 2019; Sorrells et al., 2019), arguing for a higher relevance of dormant precursors in this early stage of life rather than for elderliness. If one follows the cognitive hypothesis stated above that, after the stereotyped prenatal brain developmental program, dormant precursors are relevant to adapt and optimize the processing of inputs to the environmental needs and situation of a specific individuals, the usefulness for these dormant precursors will be eminent in the young ages during which many “first time experiences” occur, e.g., securing independent food collection, mating, predator avoidance, etc. Thus, an evolutionary advantage represented by precursor’s maturation in early adulthood could be explained by increased individualization and ontogenetic adaptation to a challenging environment as already proposed for hippocampal progenitors (Julia et al., 2013; Abrous et al., 2021). In contrast, the advantage of integrating dormant precursors thereafter during aging may be sparse under stable life conditions.

Even if the role of dormant precursors in aging is speculative, it is interesting to consider that while in mammals with short lifespan (e.g., mice) dormant precursors mature completely within a year (Rotheneichner et al., 2018), mammals with longer lifespan have precursors that can retain immaturity for decades (La Rosa et al., 2020; Sorrells et al., 2021). It is therefore tempting to hypothesize that in species with longer period of immaturity, at least in relation to their reproductive behavior, there may be an evolutionary advantage in retaining a slower pace of dormant precursor maturation befitting the slower rate of brain maturation. If so, dormant precursors may be beneficial by supporting a greater degree of adaptability in an advanced age. A crucial question to answer before addressing the matter is whether late-immature precursors can become bona fide mature neurons in the aged brain altogether. Once mechanisms that control the timing of maturation onset are understood, delaying or obliterating precursor maturation will help to understand the contribution of precursors in the aged brain.

### Pathology

At the dawn of the twentieth century, pioneers of neuroscience were convinced that the adult brain was devoid of significant regenerative potential and that adult neurons which died could not be replaced. Thus, after the frame-shifting discovery of adult neurogenesis (Altman and Das, 1965), the possibility of a regenerative potential in the adult brain was explored in the attempt to tap into the neurogenic reservoirs of the brain, cure disease, and prolong life (Shivraj Sohur et al., 2006; Santos et al., 2012; Braun and Jessberger, 2014; Rahman et al., 2021). By analogy, it is justified to question whether the dormant precursors might be suited to replace lost neurons along aging, or after brain damage or neurodegeneration. Some observations argue in favor of this hypothesis. For instance, bulbectomy was found to trigger dormant precursor maturation (Gómez-Climent et al., 2011). This brain trauma causes the loss of principal neurons in the olfactory cortex (Koliatsos et al., 2004), which in turn triggers integration of new neurons in a putative attempt to replace the dying population (Rossi et al., 2014). However, bulbectomy alters the noradrenergic signaling that, as mentioned above, controls cellular plasticity in the olfactory cortex and maybe indirectly the process of precursor maturation as well (Pohorecky et al., 1969; Vadodaria et al., 2017).

On the other side, the evidence in favor of the relevance of dormant precursors in pathology are challenged by various observations. First, dormant precursors do not proliferate in the adult brain, and thus their availability is inherently limited (Luzzati et al., 2009; Piumatti et al., 2018). Furthermore, dormant precursors are not uniformly distributed within the cerebrum, but reside in specific brain areas (Feliciano et al., 2015; König et al., 2016; La Rosa et al., 2020). Moreover, despite the absence of pathology, dormant precursors progressively mature in the healthy brain according to a staggered pattern (Rotheneichner et al., 2018; Coviello et al., 2021). Finally, to the best of our knowledge, no evidence suggests that mammalian species endowed with more dormant precursors, such as the gyrencephalic species, can deal better with brain trauma or degeneration than the lissencephalic species, and thus the dormant precursors may be simply underpowered to repair the adult brain.

Yet, pathology has undeniable effects on the maturation and can lead to an aberrant integration of adult neuronal precursors within the neurogenic regions of the brain [see for example Ceanga et al. (2019)]. It is reasonable to expect that rather than counteracting consequences of pathologies, dormant precursors may be impacted by pathological processes over long periods of time, leading to an altered integration of precursors and thereby contributing to dysfunctional brain plasticity during adulthood. However, as long as the physiological role of dormant precursors remains unknown, the consequence of their dysfunction will remain conjectural.

## How Will We Know More About the Roles of Dormant Precursors?

A necessary step toward resolving the role of matured dormant precursors in the adult brain will be to understand their connectivity. In the murine olfactory cortex, the similarity between matured precursors and other neighboring principal neurons leads to question whether the latecomers develop connectivity patterns that recapitulate those of postnatal-matured neurons (Johnson et al., 2000). Putative axonal targets of matured precursors of the olfactory cortex may be found in the olfactory bulb, the anterior olfactory nucleus, the olfactory tubercle as well as the amygdaloid, perirhinal, entorhinal, and insular cortex. Extending the same logic to other brain areas, tracing and imaging will help to unravel the matured precursor connectivity.

Unfortunately, the analysis of maturation and connectivity is so far limited to species in which a transgenic reporter system is available (Zhang et al., 2010; Rotheneichner et al., 2018). Defining function and connectivity of precursors in gyrencephalic mammals is therefore challenged by the lack of strategies to label matured precursors. In species that developed the highest degree of brain complexity, modulation of the connectivity patterns could allow for a degree of brain network plasticity previously unimaginable (Frotscher, 1992). Given the species-specific patterns of precursor distribution, developing new strategies to trace matured precursors in gyrencephalic mammals may be the key for the most crucial discoveries. Current theoretical models of adult brain network activity disregard the existence of dormant precursors and are therefore inaccurate. Understanding the final connectivity of the latter is a prerequisite to consolidate the current models.

Another crucial step toward resolving the role of matured precursors will be to actively control their maturation. Differences between the functional traits of postnatal-developed neurons and matured precursors suggest a slow pace of maturation after onset (Benedetti et al., 2019). Moreover, the staggered onset of maturation for individual cells over time and throughout the course of life constitutes a major obstacle hindering a full temporal resolution over the maturation process. Thus, at a given age, maturing precursors observed in the adult brain constitute a mixed pool at heterogeneous states of maturation (Sorrells et al., 2019; La Rosa et al., 2020). Deciphering the mechanisms that trigger or hinder the dormant precursor maturation could allow to synchronize the process and overcome the problem. Moreover, controlling the molecular pathways responsible for the maturation of dormant precursors will also allow to understand the relevant physiological and pathological signals that cause or derive from altered maturation and behaviors.

Therefore, pharmacological modulation of precursor maturation may allow to appreciate indirect effects of brain diseases leading to new therapeutic targets. In this regard, recent research on humans suggested a connection between dormant precursors in the amygdala and autism (Avino et al., 2018; Sorrells et al., 2019). Furthermore, research on animal models implies a relation between dormant precursor maturation and monoamine signaling (Vadodaria et al., 2017; Coviello et al., 2020), opening perspectives toward models of psychosis and/or depression.

## Conclusion

Compelling evidence implies that dormant precursors in the adult brain are physiologically relevant and may contribute to an overlooked form of late brain maturation. Intriguingly, our brain seems to use this resource sparingly throughout the whole course of life. To fully understand the contribution of dormant precursors integration, it will be crucial to identify the molecular mechanisms promoting or hindering maturation and the behavioral impacts.

Considering the possibility of a precursor-based contribution to learning and adaptation of input processing in the young individuals may shed a new light on educational strategies, professional development, rehabilitation, etc. In addition, the handful of dormant precursors still available up to an advanced age may also constitute a precious resource and understanding the mechanisms that promote their late integration could allow to recover every last bit of untapped potential, perhaps improving cognition and/or adaptation in the aging brain. Importantly, the number of dormant precursors is inherently limited by their non-proliferative nature and purposely promoting their integration in early life will lead to their premature exhaustion. Therefore, a comprehensive understanding of the relevance of dormant precursors in processes of brain maturation and adaptation along the different life phases constitutes a pressing need.

## Data Availability Statement

The original contributions presented in the study are included in the article/supplementary material, further inquiries can be directed to the corresponding author/s.

## Author Contributions

BB and SC-D designed and wrote the manuscript. Both authors contributed to the article and approved the submitted version.

## Conflict of Interest

The authors declare that the research was conducted in the absence of any commercial or financial relationships that could be construed as a potential conflict of interest.

## Publisher’s Note

All claims expressed in this article are solely those of the authors and do not necessarily represent those of their affiliated organizations, or those of the publisher, the editors and the reviewers. Any product that may be evaluated in this article, or claim that may be made by its manufacturer, is not guaranteed or endorsed by the publisher.
